# Immobilization of Collagen on the Surface of a PEEK Implant with Monolayer Nanopores

**DOI:** 10.3390/polym14091633

**Published:** 2022-04-19

**Authors:** Hun Kim, Yang Ho Lee, Nam Kwon Kim, Inn Kyu Kang

**Affiliations:** 1 Institute of Jeil Life Engineering, Jeil Medical Corporation, Seoul 08375, Korea; biohuny@jeilmed.co.kr (H.K.); fine.lee31@gmail.com (Y.H.L.); tstar@jeilmed.co.kr (N.K.K.); 2 Department of Polymer Science and Engineering, Kyungpook National University, Daegu 41566, Korea

**Keywords:** PEEK, orthopedics, acid treatment, surface, single-layered nanopores

## Abstract

Polyetheretherketone (PEEK) is the only polymer material that can replace titanium implants in the field of orthopedics. This is because the mechanical properties of PEEK are similar to those of bone, and PEEK has natural radiolucency, chemical stability, and sterilization resistance. Despite these advantages, PEEK has a disadvantage—that it is bio-inert. Therefore, many studies have attempted to change the bio-inertness of PEEK into bioactivity. Among them, a method of forming pores by acid treatment is attracting attention. In this study, an attempt was made to form pores on the surface of PEEK implant using a mixed acid of sulfuric acid and nitric acid. As a result, it was found that the condition when the PEEK surface is in contact with the acid is very important. That is, it was possible to form single-layered nanopores on the surface by contacting PEEK with a mixed acid under ultrasound. Additionally, by immobilizing type I collagen on the porous PEEK surface through dopamine coating, it was possible to obtain collagen-immobilized porous PEEK (P-PEEK-Col) with high compatibility with osteoblasts. This P-PEEK-Col has high potential for use as a bone substitute that promotes bone formation.

## 1. Introduction 

Orthopedic implants are widely used in clinical practice to repair or rebuild bone systems damaged by trauma or tumor resection [1]. Nevertheless, the most commonly used implant materials, titanium and titanium alloys, suffer from modulus mismatch, stress shielding, and strong interference with standard imaging modalities used to evaluate repaired bone systems [2]. As an alternative, polyetheretherketone (PEEK) is of great interest due to its elastic modulus similar to human bone, good mechanical properties, natural radiolucency, and good chemical and sterilization resistance [3,4]. However, despite many advantages, PEEK is bioinert, so it is necessary to increase its bioactivity [5,6,7]. One commonly accessible approach is to apply a bioactive coating such as hydroxyapatite, calcium silicate or bioglass to the PEEK surface [8]. Another strategy is to introduce a three-dimensional (3D) porous structure on the surface of the PEEK implant material [9]. This is because these structures mimic the structure of human bone and promote cell proliferation and differentiation functions.

Several methods for making PEEK scaffolds with porous surfaces have been reported. Ouyang et al. [10] established a porous PEEK scaffold fabrication using sulfonation methods, which exhibited better osseointegration and mechanical stability than high-density PEEK scaffolds. Landy et al. [11] fabricated superficially porous PEEK scaffolds through melt extrusion and salt leaching methods. They conducted experiments on the femoral defect model in mice using the obtained porous PEEK scaffold. As a result, it was reported that osseointegration was improved. However, these traditional techniques are difficult to precisely control pore size, porosity, or scaffold interconnectivity. Residual impurities and dead space issues are also pointed out. Therefore, the effect of pore structure on the mechanical and biological properties of porous PEEK scaffolds is not yet clear.

In recent years, additively fabricated scaffolds, also known as 3D printing, have been widely used in clinical therapy for bone repair [12]. Computer-aided 3D-printed materials easily display custom shapes, including pore size, porosity, and pore interconnections. Therefore, it is possible to regenerate bone tissue and provide conditions suitable for cell migration and proliferation according to patient-specific requirements. The effect of microstructured surfaces on cellular behavior has long been recognized, and it has recently been shown that cells can detect and respond to nanoscale signals as small as about 10 nm [13]. Microstructured (≥1 μm) or nanostructured (<1 μm or in particular <100 nm) surfaces can be created by spatially distributing biomolecules on a micro or nano scale or modifying the surface texture to micro or nano topography [14]. Immobilization of biomolecules such as hydroxyapatite [5], collagen [15], and cell growth factor [16] on the surface has been extensively studied to improve the bioactivity of PEEK. 

In addition, a three-dimensional network structure can be imparted by treatment with concentrated sulfuric acid for a certain period of time [17,18]. Since the pores formed by sulfuric acid treatment show an interconnected shape, there is a possibility that a small amount of sulfur may remain in the pores, which is harmful to cell growth and difficult to remove. Recently, Ding et al. [19] treated PEEK with a mixed acid of nitric acid and sulfuric acid to control the pore shape, thereby reducing the possibility of residual acid compounds in the implant. They also speculated that if PEEK was endowed with high hydrophilicity and chemical groups, this would greatly enhance cell adhesion and osseointegration.

In this study, we propose a novel method to form monolayer nanopore structures on the surface of PEEK implants by treating mixed acids under ultrasound. In addition, it was attempted to enhance histocompatibility by chemically covalently bonding collagen to the nanopores on the PEEK surface.

## 2. Materials and Methods

### 2.1. Materials

PEEK, as a brownish color rod (crystallinity 32%, Tg 143 °C, Tm 343 °C), was purchased from Evonik Company, Germany. A PEEK rod with a diameter of 1.0 cm was processed into a circular disk with a thickness of 1 mm using a die, and was used in the acid treatment process to form pores on the surface. Sulfuric acid (98%) and nitric acid (65%) for acid treatment of PEEK disks were used as first-class reagents of Duksan Chemical in Korea. Dopamine hydrochloride was obtained from Sigma-Aldrich (Castle Hill, NSW, Australia). Tris(hydroxymethyl)aminomethane (Tris) buffer solution (pH 8.5), 1-ethyl-3-(3-dimethylaminopropyl dicarbodiimide hydrochloride) (EDC), N-hydroxysuccinimide (NHS) were purchased from Sigma-Aldrich Chemical Company, (St. Louis, MO, USA), and used without further purification. Alizarin red staining kits were purchased from Millipore (Billerica, MA, USA). ALP staining kits were purchased from Cell Biolabs, Inc. (San Diego, CA, USA). For collagen immobilization on the PEEK surface, a concentrated solution obtained by precipitating pig-derived type I collagen with salt was used (Ubiosis Company, Seongnam-si, Korea). The cells were cultured in α-minimum essential medium (α-MEM) (Gibco BRL, Grand Island, NY, USA) then supplemented with 10% fetal bovine serum (FBS; Gibco) and 1.0% penicillin G-streptomycin at 37 °C under a 5% CO_2_ atmosphere. The culture medium was changed every other day. Mouse pre-osteoblast cells (MC3T3-E1) were purchased from Korea cells bank (Seoul, Korea) and stored in liquid nitrogen before carrying out cells seeding experiments. A 10 × 10^−3^ mmol phosphate buffer saline (PBS) solution (pH 7.4) containing 87 × 10^−3^ mmol Na_2_HPO_4_, 14 × 10^−3^ mmol KH_2_PO_4_, 131 × 10^−3^ mmol NaCl and 27 × 10^−3^ mmol KCl was purchased from Sigma-Aldrich. All other chemicals and solvents used in experimental work were high purity reagents and were purchased from Sigma-Aldrich.

### 2.2. Surface Treatment of PEEK Implants Using Mixed Acids under Ultrasound

The previously reported method [19] was modified and used to introduce nanopores on the PEEK implant surface. In this study, the effect of ultrasound on the mixed acid treatment of PEEK implants was investigated in detail. That is, the circular PEEK implant (10 mm in diameter, 1 mm in thickness) was immersed in a mixed acid (1:1) solution of 95% sulfuric acid and 65% nitric acid under ultrasound for 90 s. After taking out the sample and removing the acid on the surface for 5 min with distilled water, the PEEK surface was washed with distilled water under a mechanical stirrer or ultrasonic wave for 6 h. Finally, it was vacuum dried for 24 h and used for surface analysis. Thereafter, PEEK having pores on the surface was named P-PEEK.

### 2.3. Measurement of Pore Size and Pore Layer Thickness of PEEK Implant Surface 

After sputter coating of gold on the P-PEEK implant, an image of the surface was obtained using a field emission scanning electron microscope (FE-SEM; 400 Hitachi, Tokyo, Japan), and the pore size was measured from there. In addition, to measure the thickness of the pore layer formed on the PEEK implant surface, the P-PEEK was etched in a V-shape using a focused ion beam (FIB) [20]. After the gold was coated on the etched P-PEEK as a thin film, the thickness of the pore layer formed on the surface was measured from the FE-SEM image.

### 2.4. Measurement of Tensile and Compressive Strength of Porous PEEK Implants 

The tensile strength and compressive strength of the porous PEEK implant and non-porous PEEK implant, respectively, were measured. Tensile strength measurement was performed by preparing standard dumbbell-type specimens in accordance with Section E 5 type by the tensile strength measurement method (ASTM D638) [21] of PEEK used for orthopedic implants presented by the Korea Food and Drug Administration (KFDA). The tensile strength measurement was performed at a temperature of 24 °C and a humidity of about 35% using a model ST-1002 (Salt Co., Ltd., Incheon, Korea). Twelve PEEK samples were measured and used as a reference value. P-PEEK was measured 3 times, 12 at a time. The values obtained in each time were expressed as groups. 

### 2.5. Covalent Bonding of Collagen on Porous PEEK Implant 

**Figure 1:**
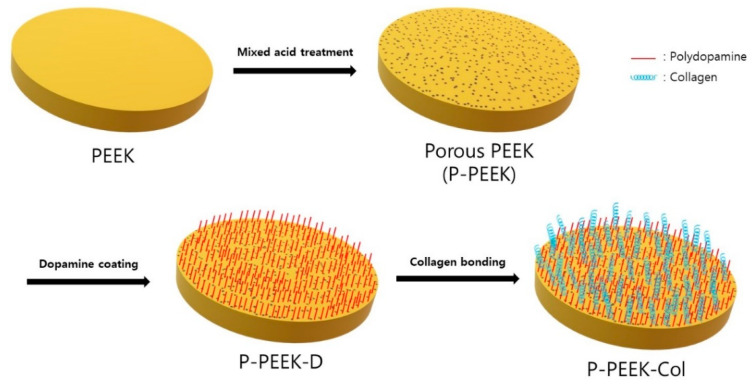
Schematic diagram showing the steps of forming pores by acid-treating PEEK surface and immobilizing collagen following dopamine coating.

Collagen physically immobilized on the PEEK implant surface is easily detached in a humid environment, whereas covalently immobilized collagen is not easily detached and is stable [22]. In this study, collagen was covalently fixed to a porous PEEK implant (Figure 1). In order to introduce functional groups to which collagen can react, PEEK disks were first immersed in dopamine Tris-buffer solution. As such, polydopamine introduced by self-assembly polymerization has been known to provide strong adhesive interactions with various materials and biomolecules including amine and thiol functional groups [22]. That is, five porous PEEK implants were immersed in Tris buffer solution (pH 8.5) containing 2.0 mg/mL dopamine and stirred at room temperature for 24 h. The resulting polydopamine-coated PEEK implant (hereinafter PEEK-D) was carefully lifted and dried at room temperature. For collagen immobilization on the PEEK surface, a concentrated solution obtained by precipitating pig-derived type I collagen with salt was used. Collagen was activated by first dissolving 0.01 wt% of collagen in a buffer solution, and reacting by adding 2.5 μg of EDC (10 mM) and 0.25 μg of NHS (25 mM) in sequence. PEEK-D was then immersed in this collagen solution and reacted at 5 °C for 48 h. Finally, it was washed with deionized water for 3 min by ultrasonic wave. In this process, most of the physically adsorbed collagen is desorbed, and the chemically covalently bonded collagen is maintained. The collagen-immobilized PEEK implant (hereinafter, P-PEEK-Col) was stored in a PBS solution (pH 7.4) at 5 °C.

### 2.6. Elemental Composition of PEEK Implant Surface by XPS 

The surface element content of pristine PEEK and dopamine- and collagen-coated PEEK implants with monolayer pores on the surface were measured using X-ray photoelectron spectroscopy (XPS, ESCA LAB VIG microtech Mt 500/1, etc., East Grinstead, UK). The XPS of the PEEK implant was subjected to qualitative and quantitative chemical analysis using the binding energy and peak intensity of elements in the irradiation spectrum [23].

### 2.7. Cell Attachment on the Porous PEEK Implant Surface

To investigate the adhesion properties of MC3T3-E1 cells on porous PEEK implant surfaces, circular PEEK implants were sterilized by UV irradiation and placed in a 24-well cell culture plate. Non-osteogenesis α-minimum essential medium (500 μL, α-MEM: Gibco, Tokyo, Japan) supplemented with 10% fetal bovine serum and 1% penicillin/streptomycin was added to each well and allowed to stand for 2 h. Then, MC3T3-E1 (5 × 10^4^ cells/mL) was added and the cells were incubated for 6 h and 3 days at 37 °C in a humidified atmosphere in the presence of 5% CO_2_. The supernatant was carefully removed, and the PEEK disks incubated with the cells were washed twice with phosphate-buffered saline and fixed with 2.5% glutaraldehyde solution for 10 min. Finally, the scaffolds were dehydrated with a critical point dryer (EMS 850 Critical Point Dryer, Hatfield, PA, USA), and the cultured cells were observed under an FE-SEM microscope (400-Hitachi, Tokyo, Japan) [15].

### 2.8. Alizarin Red and Alkaline Phosphatase Staining 

Alizarin red staining of cultured MC3T3-E1 cells was performed to evaluate the osteogenic response of cells in porous PEEK implants [24]. Briefly, MC3T3-E1 cells were cultured for 14 days in a humidified atmosphere of 5% CO_2_ at 37 °C at a cell density of 5 × 10^4^ cells/mL in circular PEEK disks placed in wells of a 24-well cell culture plate. After that, the medium was carefully aspirated and the discs were washed twice with PBS solution, and then the adhered cells were fixed at room temperature for about 15 min using a 10% aqueous formaldehyde solution. After fixation, the cells were rinsed 3 times (10 min each) with distilled water to remove residual formaldehyde. After removing excess water, 1 mL of alizarin red solution was added to each well, and the sample was incubated for 30 min to perform alizarin red staining of the cultured cells. After staining, the sample was gently washed with distilled water to remove excess alizarin red. 

Meanwhile, alkaline phosphatase (ALP) activity of MC3T3-E1 cells cultured on a porous PEEK implant was measured as follows. Sterile porous PEEK discs were placed in a 24-well cell culture plate and MC3T3-E1 cells were plated thereon at a cell density of 3 × 10^4^ cells/mL. Cells were cultured at 37 °C and 5% CO_2_ in a humidified atmosphere for 14 days. ALP staining of MC3T3-E1 cells was performed using the protocol specified by the supplier of the ALP staining kit. PEEK discs were washed with deionized water and fixed using a citrate-acetone formaldehyde fixative (25 mL citrate solution, 65 mL acetone, and 8 mL 37% formaldehyde solution) for approximately 30 s. MC3T3-E1 cells from PEEK discs were stained with an alkaline dye mixture containing 8 mL of Fast Blue RR salt solution and 0.4 mL of naphthol AS MX phosphate alkaline solution for approximately 30 min at room temperature. After staining, the PEEK discs were rinsed with deionized water for approximately 2 min to remove residual dye. After this washing, porous PEEK discs were placed in Mayer’s Hematoxylin solution for about 10 min. Digital images were captured with a light microscope equipped with an advanced camera (Axioplan 2, Carl Zeiss camera) to obtain images of alizarin red- and ALP-stained cells on PEEK samples [24]. 

### 2.9. In Vitro Cytotoxicity of Porous PEEK Implant

To evaluate whether the porous PEEK implant induces cytotoxicity, an extraction method according to ISO 10993-5:2009 was conducted. Porous PEEK implants were lysed using MEM medium supplemented with 10% serum. This eluate was applied to a uniform monolayer of fibroblasts. At the same time, three solvent controls, negative controls, and positive controls were also treated. All cells were cultured at 37 °C, 5% CO_2_ for 48 h, morphological changes and lysis of each cell were observed under a microscope, and the number of viable cells was counted.

## 3. Results 

### 3.1. Formation of Single-Layered Nanopores on the Surface of PEEK Implants

It has been reported that immersion of PEEK implants in concentrated sulfuric acid for 30 s leads to the formation of multi-pore layers of circular and circular fibrils on the surface. [17,18]. This fast reaction rate of PEEK implants with concentrated sulfuric acid can be slowed down by using mixed acids. According to a study by Ding et al. [19], micro- and nano-sized pores were simultaneously formed irregularly on the surface of PEEK implants when treated with a mixed acid under magnetic stirring for 90 s. When PEEK is treated with a mixed acid of sulfuric and nitric acid, sulfonation occurs first. Then, nitration by nitric acid proceeds using the produced aromatic sulfonic acid as a catalyst. At this time, sulfite ions are released and the PEEK surface is stabilized. In this study, forming a well-controlled nanopore structure on the surface was attempted by optimizing the conditions for treating PEEK implants with a mixed acid.

**Figure 2:**
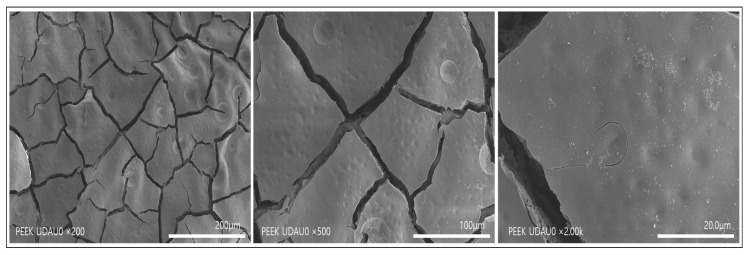
SEM images of PEEK implants treated with a mixed acid of sulfuric and nitric acid for 90 s under ultrasound and then washed with distilled water under mechanical agitation for 6 h.

Figure 2 shows the SEM image of PEEK implant treated with mixed acid for 90 s under ultrasound and washed with distilled water for 6 h under mechanical stirring. As shown in Figure 2, PEEK fragments with a width of several tens to several hundred micrometer were layered on the surface. We replaced the cleaning process with ultrasonic waves in a mechanical stirrer to remove fragments of the surface layer.

**Figure 3:**
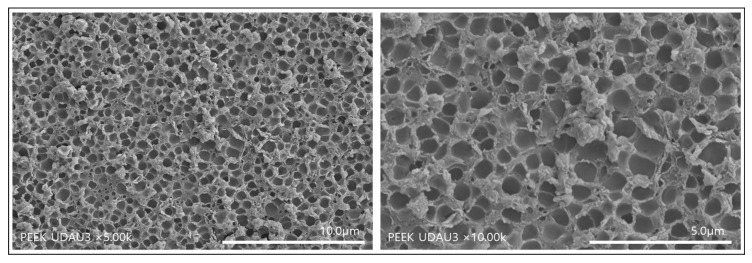
SEM images of PEEK implants at various magnifications after treatment with a mixed acid of sulfuric acid and nitric acid under ultrasound for 90 s and then washing with distilled water for 6 h under ultrasound.

Figure 3 shows SEM images formed when PEEK implants were immersed in a 1:1 ratio of sulfuric acid and nitric acid under ultrasound for 90 s and washed with distilled water under ultrasound. As shown in Figure 3, it can be seen that nanopores with a diameter of 1 μm or less are formed as a single layer. This result suggests that fragments formed on the surface of the mixed acid-treated PEEK implant can be effectively removed by ultrasonic cleaning using distilled water. 

**Figure 4:**
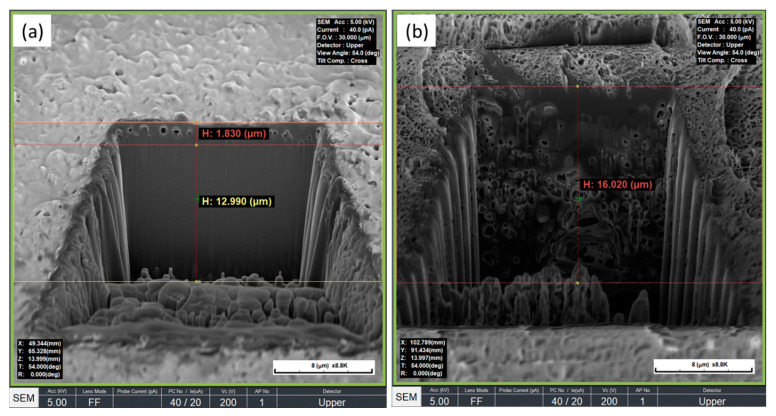
SEM image of a V-shaped etched PEEK implant (**a**) treated with mixed acid and a PEEK implant treated with sulfuric acid (**b**) with a focused ion beam (FIB).

To measure the thickness of the formed pore layer, the surface of the PEEK implant was etched in a V-shape using a focused ion beam (FIB) and then observed by SEM (Figure 4). From the image of the etched cross-section (Figure 4a), it can be seen that single-layer pores with a thickness of 0.5 to 2 μm are formed on the surface of the PEEK implant. However, in the PEEK implant treated with sulfuric acid alone (Figure 4b), various large and small pores were formed, and pores were formed from the surface of the PEEK to the depth. The depth of the pore layer was about 22.5 μm. As such, the nanopore structure of the single layer on the PEEK surface was obtained by combining the mixed acid, and ultrasonic waves were completely different from the surface pore structure generated by the treatment with sulfuric acid alone [17]. 

**Figure 5:**
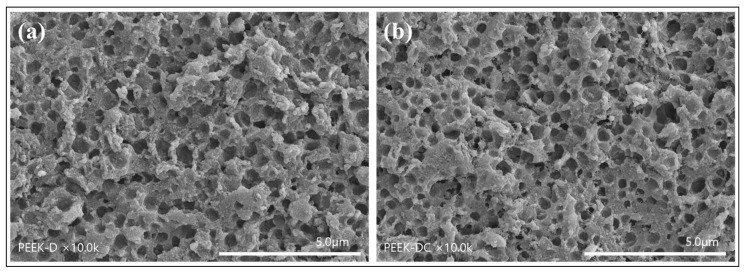
SEM images of porous PEEK coated with polydopamine (**a**) and porous PEEK sequentially immobilized with dopamine and collagen (**b**).

After polydopamine was coated on the surface of the porous PEEK implant, type I collagen was chemically covalently bonded. To investigate whether polydopamine coating and collagen immobilization affect the maintenance of nanopores on the PEEK surface, it was observed with a scanning electron microscope. As can be seen in Figure 5, it was found that the nanopores formed on the surface were well maintained even after polydopamine coating (Figure 5a) and collagen immobilization (Figure 5b). The collagen-immobilized porous PEEK is expected to play a very positive role in biocompatibility with bone tissue during implant insertion.

### 3.2. Relationship between Surface Porosity and Mechanical Properties

How acid-induced pores affect the mechanical properties of PEEK is an important concern in implant research. Table 1 shows the mechanical strength of PEEK implants having single-layer nanopores on the surface. Compared to the tensile strength and compressive strength of non-porous PEEK, the tensile strength and compressive strength of porous PEEK decreased by 1.2% and 0.29%, respectively. From these results, it was found that the effect of surface nanopores formed by acid treatment on the mechanical properties of PEEK was extremely small.

### 3.3. Immobilization of Collagen on Porous PEEK Implant Surface

**Figure 6:**
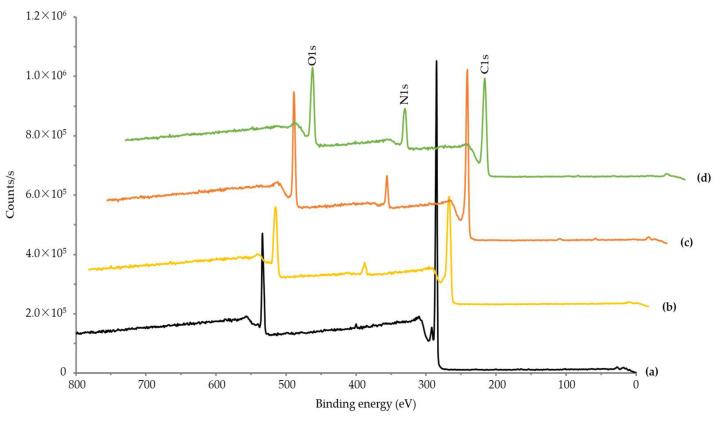
X-ray photoelectron spectroscopy of pristine PEEK (**a**), porous PEEK (P-PEEK, (**b**)), dopamine-coated PEEK (P-PEEK-D, (**c**)), and collagen-immobilized PEEK (P-PEEK-Col, (**d**)).

To confirm that dopamine and collagen were successfully introduced to the porous PEEK surface, elemental analysis of the sample surface was performed using XPS. As shown in Figure 6, C1s, O1s, and N1s appeared as central peaks in the surface-modified PEEK. The chemical compositions of the porous PEEK implants, calculated from the XPS survey scan spectra, are shown in Table 2. The N value of 4.67 in P-PEEK is due to nitration during acid treatment [19]. In addition, increasing N values to 8.64 in P-PEEK-D and 16.23 in P-PEEK-Col, respectively, indicate that dopamine and collagen were successfully introduced into the PEEK surface [15].

### 3.4. MC3T3-E1 Cell Behavior on PEEK Implants with Nanopores on the Surface

**Figure 7:**
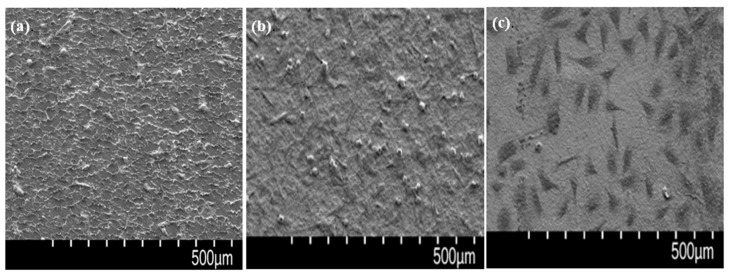
SEM images of MC3T3-E1 cell adhered to pristine PEEK (**a**), P-PEEK (**b**), and P-PEEK-Col (**c**) after 6 h culture.

After culturing the osteoblasts on the pristine PEEK, P-PEEK, and P-PEEK-Col implant for 6 h, the attached cell image was observed with SEM. The mechanically processed PEEK control (Figure 7a) had a rough surface and cells were adhered thereon. More cells were attached to the P-PEEK with the nanopore surface (Figure 7b). On the other hand, attached cells spread well on collagen-immobilized porous PEEK (P-PEEK-Col) (Figure 7c). The rapid attachment and proliferation of cells on P-PEEK-Col is due to the specific recognition between the integrin of the osteoblast membrane and the arginine-glycine-aspartic acid (RGD) sequence of immobilized collagen [25].

**Figure 8:**
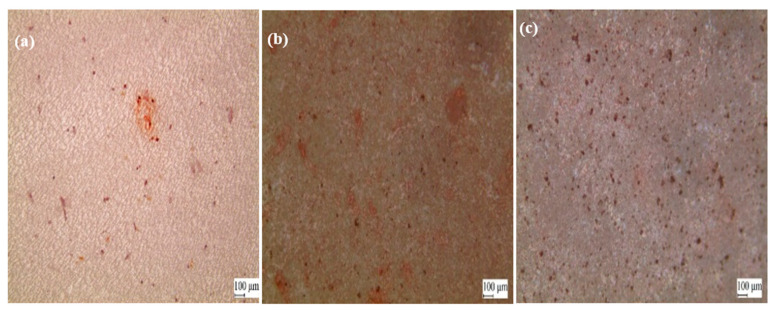
Alizarin red staining of MC3T3-E1 cells cultured on pristine PEEK (**a**), P-PEEK (**b**), and P-PEEK-Col (**c**). MC3T3-E1 cells were cultured for 14 days at 37 °C at a cell density of 5 × 10^4^ cells/mL in circular PEEK disks placed in wells of a 24-well cell culture plate. After removing the medium, the adhered cells were fixed with 10% formaldehyde aqueous solution. Thereafter, alizarin red staining was performed on the cultured cells. Digital images were captured with an optical microscope equipped with an advanced camera to obtain images of cells stained with alizarin red in PEEK samples.

Alizarin red staining reveals the transformation of undifferentiated MC3T3-E1 cells into osteoblasts and subsequent mineralization leading to bone formation [15]. Alizarin red staining in vitro can visualize the osteogenesis process by producing a red color in calcium deposits. In this study, we investigated the osteogenic properties of various PEEK surfaces in MC3T3-E1 cells using alizarin red staining (Figure 8). As can be seen in Figure 8 (red spot), the calcium-producing ability of MC3T3-E1 cells cultured on the P-PEEK (b) and P-PEEK-Col (c) surfaces was better than that of MC3T3-E1 cells cultured on the PEEK (a) surface. 

**Figure 9:**
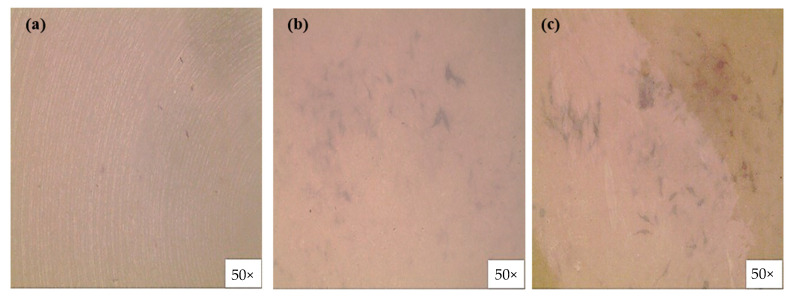
Alkaline phosphatase activity staining of MC3T3-E1 cells cultured for 14 days on pristine PEEK (**a**), P-PEEK (**b**), and P-PEEK-Col (**c**).

Collagen type I and ALP are considered early markers of osteogenic differentiation [26], whereas osteocalcin (OCN) and osteopontin (OPN) are considered late markers. In this study, we evaluated the alkaline phosphatase activity of osteoblasts cultured on porous PEEK (Figure 9a) and collagen-immobilized porous PEEK (Figure 9c). When MC3T3-E1 cells are stained with an ALP kit and a purple image appears, it means that the expression of alkaline phosphatase is improved. As can be seen in Figure 9, almost no ALP activity was seen in the non-porous PEEK (a). However, ALP activity was clearly increased in porous PEEK (Figure 9b) and collagen-immobilized PEEK (Figure 9c). The increase in ALP activity of MC3T3-E1 cells indicates that the nanopores on the PEEK surface and the introduced collagen improved the expression of alkaline phosphatase, which is important for osteogenic differentiation and bone formation.

### 3.5. In Vitro Cytotoxicity of Porous PEEK Implant

In the cells contacted with the eluate of P-PEEK, a uniform monolayer was maintained after incubation, and there were almost no toxic cells. Therefore, it can be seen that the eluate of P-PEEK has no reactivity to cultured cells. In addition, as a result of quantitative analysis by counting live cells, it was confirmed that the eluate of P-PEEK had no effect on the number of live cells. The solvent control group and the negative control group did not show toxicity to the cultured cells, and the positive control group caused cytotoxicity in more than 75% of the total cells as expected.

## 4. Discussion

PEEK is attracting attention as a biomaterial that can replace titanium implants because its elastic modulus is similar to human bone and has excellent chemical and sterilization resistance. However, since PEEK is bio-inert, it is necessary to impart bioactivity. The most well-known method for converting the bio-inertness of PEEK into bioactivity is to introduce pores on the surface. To realize this, 3D printing [27] and processing [28] methods have been actively carried out. Porous PEEK has also been prepared by injection molding using NaCl crystals with a diameter of 200 to 450 μm. In this case, the pores formed have an interconnected structure and the pore sizes in the range of several hundred micrometers significantly reduce the mechanical properties of PEEK, particularly the percent strain at break [29].

As a preliminary study, we tried to form pores on the PEEK surface using various concentrations of sulfuric acid. However, the etching rate of PEEK by sulfuric acid was too fast to control pore formation. In addition, the obtained pore shapes were all interconnected pore shapes [17], and this was not the pore shape we wanted. In addition, it was found that pores were not formed when PEEK was treated using only nitric acid. Su et al. [28] developed a sulfuric acid-treatment strategy to create microporous architectures onto the filaments of the additively-manufactured PEEK lattice scaffolds. The sulfuric acid treatment time in the range of 30–45 s was found to facilitate the formation of uniform microscale pores throughout the printed PEEK lattice scaffolds and simultaneously have slight effect on mechanical properties. Biological results showed that the presence of microscale pores on the additively-manufactured PEEK lattice scaffolds significantly improved the spreading, proliferation, and calcium deposition of bone-specific cells in comparison with the untreated PEEK lattice scaffolds. In spite of many efforts, there have been no reports of research results that show little mechanical loss while maintaining bioactivity by introducing nanopores on the PEEK surface.

In this study, PEEK implants were treated under ultrasound using sulfuric acid and nitric acid in a 1:1 ratio. The obtained surface pores exhibited monolayered pores with a diameter of 1 micrometer or less and a depth of 0.5 to 2 μm (Figure 3 and Figure 4). Table 1 shows the comparison between the mechanical strength of the PEEK implant having a single layer of nanopores on the surface and the mechanical strength of the non-porous PEEK implant. The PEEK implant with a single layer of nanopores on the surface maintained 98.8% tensile strength and 98.7% compressive strength compared to non-porous PEEK. From these results, it can be seen that the PEEK implant having a single layer of nanopores on the surface prepared in this study has almost negligible loss of mechanical properties due to pore formation. Sagomonyants et al. [30] compared the behavior of osteoblasts with rough titanium using machined or injection molded PEEK. They reported that although differences in human osteoblast responses were found in the various PEEK samples, in general, implantable grade PEEK was comparable to the bone-forming ability of rough titanium in vitro. 

Surface immobilization of collagen is known as a method of increasing the cell compatibility of PEEK implants. The mussel-inspired polydopamine coating technology provides strong adhesive interactions with a variety of materials and biomolecules containing amine and thiol functional groups [22]. Kwon et al. manufactured collagen-immobilized PEEK by covalently bonding type I collagen to polydopamine-coated PEEK [15]. The amount of collagen introduced to the surface can be known by checking the N value using XPS. As can be seen from Table 2, when collagen was introduced to the porous PEEK surface (P-PEEK-Col), the N value was 16.23. This value is much larger than the N value of 10.7 obtained when collagen was introduced on a non-porous surface [15]. This is because the specific surface area of the nanopore surface has been remarkably improved and the number of reaction sites with collagen has increased. Another important reason that we were able to introduce a large amount of collagen to the porous PEEK surface is because a concentrated solution obtained by precipitating pig-derived type I collagen with salt was used. However, the collagen type I used herein is preferably extracted from the skin of a transformed pig so as not to cause hyperacute, acute vascular, and cell-mediated immune rejection in humans.

Many attempts have been made to alter the surface function to enhance the adhesion, growth, and differentiation of osteoblasts on PEEK implants. Wu et al. attempted to use the covalently coating of phosphorylated gelatin loaded with bone morphogenetic protein 2 (BMP-2) on hydroxylated micro-porous PEEK films for enhancing the biological activity [31]. Torstrick et al. investigated the effect of pore size on the PEEK surface on the proliferation and differentiation of osteoblasts [29]. As a result, cells cultured on surface porous PEEK (regardless of pore size) displayed a more differentiated phenotype than cells cultured on smooth PEEK. All surface porous PEEK groups had greater EdU DNA binding than smooth non-porous PEEK, indicating increased cell proliferation. On the other hand, there is a report that the compatibility with osteoblasts is improved when collagen is immobilized on a porous support [32].

In this study, it was possible to form single-layer nanopores on the surface of PEEK implants by treating them with a mixed acid of sulfuric acid and nitric acid under ultrasound. The loss of mechanical properties of PEEK implants with monolayer nanopores was almost negligible when compared to non-porous PEEK implants (Table 1). In addition, it was possible to introduce a large amount of collagen to the nanopore PEEK implant, and it was confirmed that the nanopores on the surface were maintained even after the collagen was introduced. As such, the P-PEEK-Col implant promotes the adhesion of osteoblasts (Figure 7) and further promotes the differentiation of osteoblasts (Figure 8 and Figure 9), suggesting the potential as an implant to promote bone regeneration.

## Figures and Tables

**Table 1:** Mechanical strength of P-PEEK implant treated with a mixed acid of sulfuric acid and nitric acid under ultrasound for 90 s *.

Samples	Tensile Strength (kgf/mm^2^)	Compressive Strength (N)
PEEK	15.078 ± 0.085	15,168.08 ± 0.02
P-PEEK-1	14.913 ± 0.200	15,130.09 ± 0.10
P-PEEK-2	14.976 ± 0.506	15,123.93 ± 0.03
P-PEEK-3	14.897 ± 0.203	15,125.50 ± 0.04

* Twelve PEEK samples were measured and used as a reference value. P-PEEK was measured 3 times, 12 at a time. The values obtained in each time were expressed as groups such as P-PEEK-1, 2, 3.

**Table 2:** Atomic percentages of PEEK, P-PEEK, P-PEEK-D, and P-PEEK-Col implants calculated from the survey scan spectra.

Substrates	Atomic Percent of Elements (%)			
	C	O	N	S
PEEK	84.95	14.38	0.67	-
P-PEEK	75.07	20.26	4.67	-
P-PEEK-D	71.68	19.67	8.64	-
P-PEEK-Col	63.85	19.78	16.23	0.15

## Data Availability

Not applicable.
